# The Use of DNA Barcoding in Identification and Conservation of Rosewood (*Dalbergia* spp.)

**DOI:** 10.1371/journal.pone.0138231

**Published:** 2015-09-16

**Authors:** Ida Hartvig, Mihaly Czako, Erik Dahl Kjær, Lene Rostgaard Nielsen, Ida Theilade

**Affiliations:** 1 Forest Genetics and Diversity, Department of Geosciences and Natural Resource Management, University of Copenhagen, Frederiksberg, Denmark; 2 Department of Biological Sciences, University of South Carolina, Columbia, South Carolina, United States of America; 3 Global Development, Department of Food and Resource Economics, University of Copenhagen, Frederiksberg, Denmark; Field Museum of Natural History, UNITED STATES

## Abstract

The genus *Dalbergia* contains many valuable timber species threatened by illegal logging and deforestation, but knowledge on distributions and threats is often limited and accurate species identification difficult. The aim of this study was to apply DNA barcoding methods to support conservation efforts of *Dalbergia* species in Indochina. We used the recommended *rbcL*, *matK* and ITS barcoding markers on 95 samples covering 31 species of *Dalbergia*, and tested their discrimination ability with both traditional distance-based as well as different model-based machine learning methods. We specifically tested whether the markers could be used to solve taxonomic confusion concerning the timber species *Dalbergia oliveri*, and to identify the CITES-listed *Dalbergia cochinchinensis*. We also applied the barcoding markers to 14 samples of unknown identity. In general, we found that the barcoding markers discriminated among *Dalbergia* species with high accuracy. We found that ITS yielded the single highest discrimination rate (100%), but due to difficulties in obtaining high-quality sequences from degraded material, the better overall choice for *Dalbergia* seems to be the standard *rbcL*+*matK* barcode, as this yielded discrimination rates close to 90% and amplified well. The distance-based method TaxonDNA showed the highest identification rates overall, although a more complete specimen sampling is needed to conclude on the best analytic method. We found strong support for a monophyletic *Dalbergia oliveri* and encourage that this name is used consistently in Indochina. The CITES-listed *Dalbergia cochinchinensis* was successfully identified, and a species-specific assay can be developed from the data generated in this study for the identification of illegally traded timber. We suggest that the use of DNA barcoding is integrated into the work flow during floristic studies and at national herbaria in the region, as this could significantly increase the number of identified specimens and improve knowledge about species distributions.

## Introduction

Conservation of threatened species is an essential part of reaching the target of the Convention on Biological Diversity 2020 on improving the status of global biodiversity [1]. The first crucial step in conserving and managing threatened species is correct identification and delimitation of the target species [2]. Identification of plant species traditionally relies on morphological characters of especially reproductive parts, such as flowers and fruits, which for trees can be time consuming to access and only present during parts of the year. Accurate identification in species-rich or taxonomically complex groups also typically requires expert knowledge, which is not always available, especially in tropical areas [3, 4]. Correct taxonomical identification of endangered tropical tree species is thus often difficult. For threatened species, whose trade is regulated by the Convention on International Trade of Endangered Species (CITES), correct identification is crucial for the enforcement of the regulations and future conservation of the species. The identification process can be problematic, especially if similar non-threatened species also appear in the trade, and if only parts of the plant are being traded (e.g. wood).

A potential method to meet these identification challenges is DNA barcoding, which is the identification of species by a short universal DNA sequence, that exhibits a sufficient level of variation to discriminate among species [5]. The key advantage of DNA barcoding is that once a solid reference database has been established, the method does not require expert taxonomic knowledge in order to identify specific samples. Further, identification can be done with small tissue samples from virtually any part of the organism, does not require reproductive material, and the identification process is in general fast and reproducible. A limitation of the method is that no single universal DNA region that can be used across all taxonomic groups have been identified. While many DNA barcoding studies in animals have achieved high rates of species discrimination using a single region, COI (see e.g. [6–8]), for plants it has proven necessary to use a combination of regions to obtain sufficient discrimination success [9–12]. Further, within taxonomic groups it is not always possible to discriminate between recently diverged species (e.g. [13]). However, since the introduction in 2003, the method is now widely applied in plant studies. First of all DNA barcoding can be used as a tool for identifying species that are difficult to identify based on morphological characters, or be used as a supporting taxonomic tool in delimitation and description of problematic species [14, 15]. The technique can also prove valuable for accurate species identification as the important first step in conservation plans for threatened species [16]. An important use of DNA barcoding is in wildlife forensics, where it has shown ability to discriminate CITES-species from similar non-threatened species [17, 18]. Apart from identifying target species, DNA barcoding can also be applied in floristics. Constructing a DNA barcoding reference library of entire local floras can allow for fast and effective floristic analyses without expert knowledge [19, 20], or even be a method of estimating species richness in a taxonomically poorly known flora [21]. There is also a huge potential for application of DNA barcoding to the vast collections at herbaria and Natural History Museums, which could serve as excellent reference databases and help identify the many un-identified specimens present in most collections, as well as identify new collections [22]. This would contribute significantly to improved knowledge about distribution patterns of each species.

DNA barcoding thus has the potential of becoming an important supporting tool for conservation and biodiversity assessments in areas with a high number of plant species, a shortage of expert taxonomists, and limited descriptions of the flora.

In the present study we address the applicability of DNA barcoding to support conservation efforts of *Dalbergia* species in Cambodia, Laos and Vietnam (Indochina in the narrow sense). As part of the Indo-Burma hotspot, the Indochina area is characterized by high levels of endemic biodiversity under threat of extinction [23] and the flora of many areas remains yet to be fully explored (see e.g. [24]). DNA barcoding could contribute to generate knowledge on taxonomy and distribution of threatened species, and help increase identification rates in floristic investigations.

The pantropical genus *Dalbergia* L.f. (Fabaceae: Papilionoideae) is an example of a taxonomic plant group that is in need of better identification tools in order to set up proper conservation guidelines. The genus consists of shrubs, lianas, and trees with around 250 species in total, and Indochina represents one of its centers of diversity with approximately 30–45 species [25–27]. A number of *Dalbergia* trees possesses high-quality timber known as e.g. rosewood, blackwood, cocobolo or palisander and the wood is used for construction works, fine furniture, music instruments etc. [27]. Examples of economically important species include African blackwood (*D*. *melanoxylon*), Brazilian rosewood (*D*. *nigra*) and Thailand rosewood (*D*. *cochinchinensis*). Many *Dalbergia* species are also used in traditional medicine for various purposes, and have been subject to phytochemical studies [28]. Overexploitation and illegal logging have severely decreased population sizes of many of the species and several timber species have been included in CITES, including *D*. *nigra* and *D*. *cochinchinensis*. Fifty-six species of *Dalbergia* are currently listed in the endangered categories at the IUCN red list [29], although this list is in great need of update, also taxonomically.

The timber of the *Dalbergia* species is often difficult to identify by wood anatomy alone [30, 31], which limits the enforcement of CITES regulations, as timber identification is a technical requirement for monitoring and controlling trade. It has recently been shown that barcoding has the potential to overcome this limitation, as it was possible to discriminate between two *Dalbergia* timber species that were morphologically indistinguishable [31]. Many species of *Dalbergia* in the Indochina area are also not easily recognized in the field, even with fruits or flowers present (J. F. Maxwell, pers. comm.). The complications are increased by the lack of a complete updated taxonomic revision, and although a regional revision exists for the Indochina area, synonyms and old names are still in use. An example is the threatened *D*. *oliveri*, which produces valuable timber and is subject to illegal logging in Indochina [32, 33]. In the latest revision of *Dalbergia* in Indochina from 1997, Niyomdham *et al*. [25] included *D*. *bariensis*, *D*. *dongnaiensis*, *D*. *mammosa* and *D*. *duperreana* as synonyms for *D*. *oliveri*. This has been accepted to some extent (e.g.[34]) although the name *D*. *bariensis* is still widely used in Cambodia. Also, in the first and only attempt of a molecular phylogeny for the whole genus Vatanparast *et al*. [35] maintained *D*. *dongnaiensis* as a separate species, and the synonyms also still exist on the IUCN red list as threatened in variable categories [29]. Solving taxonomic issues like this is essential for establishing effective conservation plans for the many threatened *Dalbergia* species in Indochina.

We apply DNA barcoding methods to 31 *Dalbergia* species, with a focus on species from the Indochina region. We use the coding plastic regions *rbcL* and *matK*, as recommended by Plant Working Group of the Consortium for the Barcode of Life (CBOL) as the core barcode for land plants [12]. As these markers not always yield high discrimination rates, we also apply the highly variable ITS region, which seems to be a valid supplement to the core barcode [36, 37].

We also address the efficacy of different analytic approaches of DNA barcode data. As a supplement to the standard distance-based methods of evaluating barcode performance [38, 39], we apply a machine learning approach, using the programs BLOG and WEKA [40, 41]. In a comparison of different barcoding analyses methods, Van Velzen *et al*. [42] found that the character-based BLOG had the highest identification rate over similarity and tree-based methods. WEKA was used by Weitschek *et al*. [43] to apply four different supervised classifiers on DNA barcode data, and they found that these methods could be used with success on barcoding datasets, reaching higher classification success than more classical barcoding methods as neighbor-joining (NJ), BLAST and nearest-neighbor.

The specific aims of this study are to i) establish a reference library for *Dalbergia* using the recommended *rbcL*+*matK*+ITS barcode, ii) use this barcode to infer on the taxonomy of the sampled *Dalbergia* species, iii) test the discrimination ability of the chosen markers, using both traditional distance-based methods as well as machine learning-based approaches, iv) apply the method on unidentified *Dalbergia* samples, including cambium/bark and wood samples, to explore how the method could be used in situations where identification by morphological characters is uncertain or not possible at all. We have a special focus on the threatened timber species *D*. *oliveri* and *D*. *cochinchinensis* in order to address the taxonomic confusion around *D*. *oliveri*, and the potential application of DNA barcoding for CITES identification of *D*. *cochinchinensis*. We discuss the findings and relate it to requirements needed before this tool can be incorporated as a standard procedure in e.g. CITES enforcement, identification of specimens at local herbaria or in floristics.

## Materials and Methods

### Sampling of specimens

A total of 95 *Dalbergia* and two *Machaerium* Pers. specimens were included in the study.

For the majority of samples, tissue material for DNA extraction was obtained from herbaria (AAU, C, E, K and L) or from a living collection belonging to one of the authors (M. Czako, University of South Carolina, USA). A few samples were obtained from field studies in Cambodia, Laos and Vietnam, with the necessary permissions from relevant authorities (Cambodia: Forestry Administration, Ministries of Agriculture, Forestry and Fisheries, Laos: Ministry of Science and Technology and provinces of Khammouane and Bolikhamsay, and Vietnam: Center for Biodiversity and Biosafety, Institute of Agricultural Genetics, and Cat Tien National Park). One sample was obtained from a commercial company. All *D*. *cochinchinensis* samples were obtained prior to the inclusion of the species in CITES. S1 Table gives voucher information for all samples.

Specimens were checked for synonyms and possible misidentifications, which led to new names for eight of the specimens (see S2 Table for details). Naming of specimens from Indochina followed Niyomdham *et al*. [25], except for *D*. *foliacea* Wall. ex. Benth, which is not an accepted name in either Genbank [44] nor The Plant List database [45], and *D*. *foliacea* was therefore included in *D*. *rimosa* Roxb. following Gagnepain [46] and Thothathri [47].

After this treatment, the 95 *Dalbergia* specimens represented 31 species (Table 1) and 14 unidentified specimens. Four species were from America, two from Africa (incl. Madagascar), and the remaining were from Asia, primarily Indochina (Table 1). The selection covered 19 of the 29 species treated in Flora of Cambodia, Laos and Vietnam [25]. Whenever possible, species were represented by two to five specimens, but for 11 species, sequences were only obtained successfully for one specimen. *Dalbergia oliveri* and *D*. *cochinchinensis* were represented by eight and six specimens, respectively. The two *Machaerium* species (*M*. *lunatum* (L.f.) Ducke and *M*. *salvadorense* (Donn.Sm.) Rudd) were included as outgroup for the phylogenetic analysis [48].

**Table 1:** List of *Dalbergia* species included in this study.

Taxon	Continent	Habit	No. of specimens
*D*. *assamica* Benth	Asia	Tree^[^ ^25^ ^]^	3
*D*. *benthamii* Prain	Asia	Climbing shrub^[^ ^49^ ^]^	1
*D*. *cana* Graham ex Kurz	Asia	Tree^[^ ^25^ ^,^ ^47^ ^]^	2
*D*. *candenatensis* (Dennst.) Prain	Asia	Climbing shrub/woody liana^[^ ^25^ ^,^ ^47^ ^]^	4
*D*. *cochinchinensis* Pierre	Asia	Tree^[^ ^25^ ^]^	6
*D*. *cultrata* Graham ex Benth.	Asia	Tree^[^ ^25^ ^,^ ^47^ ^]^	4
*D*. *dyeriana* Prain ex Harms	Asia	Woody liana^[^ ^25^ ^,^ ^49^ ^]^	1
*D*. *ecastaphyllum* (L.) Taub.	America	Scandent shrub/robust shrub/small tree^[^ ^50^ ^]^	2
*D*. *entadoides* Pierre	Asia	Climbing shrub^[^ ^25^ ^]^	1
*D*. *hancei* Benth.	Asia	Woody liana^[^ ^25^ ^,^ ^49^ ^]^	2
*D*. *horrida* (Dennst.) Mabb. *var*. *glabrescens* (Prain) Thoth. & Nair.	Asia	Climbing shrub/woody liana^[^ ^25^ ^,^ ^47^ ^]^	1
*D*. *hupeana* Hance	Asia	Tree^[^ ^49^ ^]^	2
*D*. *lanceolaria* Linne F.	Asia	Tree^[^ ^25^ ^,^ ^47^ ^]^	3
*D*. *latifolia* Roxb.	Asia	Tree^[^ ^47^ ^]^	1
*D*. *melanoxylon* Guill. & Perr.	Africa	Tree/shrub^[^ ^51^ ^]^	1
*D*. *mimosoides* Franch.	Asia	Shrub^[^ ^49^ ^]^	1
*D*. *miscolobium* Benth.	America	Shrub/small tree^[^ ^50^ ^]^	1
*D*. *monetaria* L.f.	America	Scandent tree/shrub/robust liana^[^ ^50^ ^]^	2
*D*. *nigrescens* Kurz	Asia	Tree^[^ ^25^ ^]^	4
*D*. *odorifera* T.C. Chen	Asia	Tree^[^ ^49^ ^]^	2
*D*. *oliveri* Gamble ex Prain	Asia	Tree^[^ ^25^ ^,^ ^47^ ^]^	7
*D*. *ovata* Graham ex Benth.	Asia	Small tree^[^ ^25^ ^,^ ^47^ ^]^/liana-like shrub^[^ ^25^ ^]^	2
*D*. *pinnata* (Lour.) Prain	Asia	Climbing shrub/small tree^[^ ^25^ ^,^ ^47^ ^]^	3
*D*. *rimosa* Roxb.	Asia	Climbing shrub^[^ ^25^ ^,^ ^47^ ^,^ ^49^ ^]^/erect shrub^[^ ^47^ ^,^ ^49^ ^]^/tree^[^ ^25^ ^,^ ^49^ ^]^	5
*D*. *sericea* G. Don	Asia	Tree^[^ ^25^ ^,^ ^47^ ^]^	1
*D*. *sissoo* Roxb.	Asia	Tree^[^ ^47^ ^]^	3
*D*. *stipulacea* Roxb.	Asia	Climbing shrub/woody liana^[^ ^25^ ^,^ ^49^ ^]^/small tree^[^ ^49^ ^]^	4
*D*. *subcymosa* Ducke	America	Climbing shrub^[^ ^50^ ^]^	1
*D*. *trichocarpa* Baker	Africa	Tree^[^ ^52^ ^]^	2
*D*. *velutina* Benth.	Asia	Climbing shrub^[^ ^25^ ^,^ ^47^ ^]^/woody liana^[^ ^25^ ^]^	4
*D*. *volubilis* Roxb.	Asia	Climbing shrub^[^ ^25^ ^,^ ^47^ ^]^/woody liana^[^ ^25^ ^]^	2

### Molecular methods

Total genomic DNA was extracted from leaves, twigs, seeds, pods, cambium/bark or sapwood samples, using slightly modified CTAB protocols [53] (full protocols can be obtained from the authors).

PCR amplifications of *rbcL*, *matK* and ITS were carried out on a Gene Amp 2700 (Applied Biosystems, USA) with Qiagen Taq PCR Master Mix kit (Qiagen, Sweden), using the manufacturer’s instructions, except that the reaction volume was increased to 50 μL. Primer sequences are listed in S3 Table. The PCR conditions for *rbcL* was 94°C for 3 min, followed by 30 cycles of 94°C for 1 min, 54°C for 1 min and 72°C for 1min, and a final step at 72°C for 10 minutes. For *matK*, the PCR conditions were 1 min at 94°C, 30 cycles of 94°C for 30 s, 54°C for 20 s and 72°C for 50 s, followed by 72°C for 5 min. For ITS, the PCR conditions were 5 min at 94°C, 35 cycles of 94°C for 1 min, 53°C for 1 min and 72°C for 1min, followed by 72°C for 7 minutes. If samples did not amplify with these conditions, annealing temperature was lowered by 1–2°C and number of cycles increased to 35 (for *rbcL* and *matK*) or 40 (for ITS).

PCR products were purified and sequenced by Macrogen Inc. (Seoul, Korea), using the same primers as for amplification.

### Data analysis

Sequences were edited and assembled in Geneious 6.0.5 to 7.1.7 (Biomatters Inc., USA). All sequences were deposited in Genbank [44] and given accession numbers (S1 Table). The edited sequences for each gene were aligned separately with Clustal W [54] as implemented in Geneious 7.1.7 (default settings), and trimmed for primer sequences in both ends. After initial alignment with Clustal W, the alignment was manually adjusted, if needed, following the principles described in Kelcher & Clark [55]. Alignments can be obtained from the author by request.

Alignments for the three barcoding regions were concatenated in Mesquite [56] to produce different combinations of datasets with variable number of taxa included for the different analyses.

### Tree-based analyses (Maximum parsimony and Neighbour joining)

To infer on the taxonomy of the sampled *Dalbergia* species, a phylogenetic analysis were conducted on the *rbcL*+*matK*+ITS dataset, including all known *Dalbergia* specimens and the two *Machaerium* species, but excluding the 14 unknown *Dalbergia* sp. (n = 83).

The analysis was performed in PAUP* vers. 4.0b10 [57] using parsimony as the optimality criterion. Uninformative characters were excluded, gaps were treated as missing data and all characters were equally weighted and unordered. The two *Machaerium* specimens were defined as outgroup species.

An initial heuristic search was carried out with 1000 replicates, holding maximum 10 trees at each step. Random stepwise addition was used for the starting tree in each replicate and branch swapping was performed by tree-bisection-reconnection (TBR). Branches with maximum length equal to zero were collapsed. A second search was performed by TBR swapping on the trees found in the first round, holding up to 15000 trees and swapping to completion.

Branch support was assessed by bootstrapping [58], using 2000 bootstrap replicates and the same settings as in the heuristic search, but with only 10 random stepwise addition sequences per replicate. Only groups that appeared in >50% of the trees were retained.

As a method of assigning the unknown *Dalbergia* specimens to species, a Neighbour-joining (NJ) tree was constructed using the *rbcL*+*matK*+ITS dataset and included all known and unknown *Dalbergia* samples, but excluded the *Machaerium* specimens (n = 95).

The NJ tree was constructed in PAUP*, using uncorrected *p*-distance [59] as genetic distance measure and setting negative branch lengths to zero. No outgroup was used. If the *Dalbergia* sp. specimen in question was found within a cluster consisting exclusively of two or more specimens of the same known species, the *Dalbergia* sp. was accepted as member of that particular species. Otherwise, it was regarded as not identified/unknown.

### Barcoding analyses

We compared the identification success of different barcoding analyses using the programs TaxonDNA, BLOG and WEKA. We performed the analyses on all combinations of barcode regions, alone or in combination of two or three regions, giving a total of seven barcodes (Tables 2 and 3). Only *Dalbergia* specimens represented by two or more specimens were included, and specimens that failed amplification for one of the three regions, were excluded from any analysis concerning the given region. This meant that numbers of specimens and species varied between the barcoding datasets evaluated (Table 2). Identification of the unknown *Dalbergia* was conducted with the *rbcL*+*matK*+ITS barcode data set only, except for two samples (*Dalbergia* sp. Cambodia1 and *Dalbergia* sp. Thailand5), where the *rbcL*+*matK* barcode was used because of amplication failure of ITS.

**Table 2:** Characteristics of the seven barcodes for *Dalbergia* spp. evaluated in this study.

Barcode	No. of specimens/No. of species	Alignment length (bp)	Mean intraspecific distance (range)	Mean interspecific distance (range)
***rbcL***	70/21	607	0.0010 (0–0.0070)	0.0068 (0–0.0181)
***matK***	68/21	775	0.0019 (0–0.0144)	0.0141 (0–0.0310)
**ITS**	56/17	662	0.0134 (0–0.0650)	0.1110 (0–0.1887)
***rbcL*+*matK***	67/21	1382	0.0013 (0–0.0084)	0.0110 (0.0008–0.0246)
***rbcL*+ITS**	55/17	1269	0.0077 (0–0.0351)	0.0600 (0–0.1041)
***matK*+ITS**	53/17	1437	0.0072 (0–0.0334)	0.0570 (0.0021–0.0930)
***rbcL*+*matK*+ITS**	52/17	2044	0.0055 (0–0.0251)	0.0420 (0.0015–0.0686)

Intra- and interspecific distances calculated using uncorrected *p*-distances between all sequence pairs.

**Table 3:** Specimen identification rates in % (correctly identified/misidentified/not identified) for *Dalbergia* spp. using six different classification methods, for each of the seven barcodes.

Barcode	TaxonDNA	BLOG	Naïve Bayes	SMO	J48	Jrip
***rbcL***	40/6/54	43/9/48	63/37/0	60/40/0	54/6/0	44/56/0
***matK***	81/13/6	81/5/24	79/21/0	81/19/0	56/4/0	57/43/0
**ITS**	89/0/11	65/23/12	88/12/0	93/7/0	61/39/0	52/48/0
***rbcL*+*matK***	87/10/3	86/5/9	78/22/0	88/12/0	63/37/0	55/45/0
***rbcL*+ITS**	89/4/7	65/6/29	85/15/0	93/7/0	65/35/0	62/38/0
***matK*+ITS**	100/0/0	65/12/23	87/13/0	94/6/0	60/40/0	57/43/0
***rbcL*+*matK*+ITS**	100/0/0	65/6/29	83/17/0	94/6/0	60/40/0	46/54/0

TaxonDNA: Best close match results. Not identified rates are summed over the “Ambiguous” and “No match” categories, see S4 Table for details. BLOG: percentage correct classification for test file, using 90% slicing at species level. Naïve Bayes = Bayesian, SMO = Support vector machine, J48 = decision tree, Jrip = rulebased, all four classification methods in WEKA, tested with 10-fold crossvalidation. See materials and methods for details on the analyses.

### Distance-based analyses (TaxonDNA)

Uncorrected *p*-distances between all sequence pairs were calculated in TaxonDNA/SpeciesIdentifier 1.7.8 [38] and used to calculate mean and range of intraspecific and interspecific distances for the seven barcodes. Uncorrected *p*-distances was chosen over the otherwise widely used K2P distance measure because there is no need for a complex model for distance measures when analyzing closely related sequences [59], and K2P seems not to be the first choice if any models should be applied [60, 61].

The ideal barcode for species identification should exhibit a ‘barcode gap’, where the minimum interspecific distance is larger than the maximum intraspecific distance within any species [39, 62]. However, as coalescent depth may vary among species, a global overlap between intra-and interspecific distances might not interfere with identification success [63]. A more accurate way is thus to evaluate the local barcoding gap, and for each species plotting the distance to the nearest non-conspecific against the distance to the furthest conspecific (e.g. [64, 65]). Hence, to evaluate the presence or absence of a local barcode gap, the maximum intraspecific and minimum interspecific distances for each sequence were found using the `extreme pairwise’ function in TaxonDNA. For each species, the maximum intraspecific distance was then plotted against the minimum interspecific distance, with a 1:1 slope representing no local barcoding gap.

TaxonDNA was also used for evaluating the specimen identification success for the seven barcodes using the `best close match’ function of the program. This method is equivalent to the nearest neighbor-method [42], and compares each sequence (query) to all other sequences (references) in the dataset, and assigns the query to the species with the reference sequence with the lowest distance to that query sequence. If multiple species have equally small distance matches, the result is considered ambiguous. If the distance to the most similar sequence(s) is outside a certain threshold level, the query sequence is classified as no match. The threshold used in these analyses was computed for each dataset during the pairwise distance analyses, and can be seen in S4 Table.

The *Dalbergia* sp. specimens were tested against the *rbcL*+*matK*+ITS barcode (*rbcL*+*matK* for the two samples that lacked the ITS sequence) using the `query against sequences’ function and applying same conditions as in the `best close match’ analysis. If the distance to the nearest reference sequences was above the threshold used in the `best close match’ analysis, the specimen was considered not identified.

### Machine-learning approach (BLOG and WEKA)

We explored the feasibility of the recently published machine-learning approaches to assign specimens to species [40, 43], in order to compare them with the traditional methods described above. DNA barcoding analysis can be viewed as a classification problem: given a *reference* data set composed of DNA barcode sequences of known species, and a *query* data set with sequences of unknown species, how can the unknown sequences be recognized as a given species present in the *reference* data set? [40, 43]. This problem can be addressed by a machine learning approach, where a classification model (different models can be applied) is build based on the *reference* dataset, where after the classification model is applied to the *query* data set [43]. The *query* data set can contain unknown or known species, where the latter will allow for verification of the classification model. If only one dataset is provided, then it can be randomly divided into *reference* and *query* data set to test the efficacy of the model [43].

Two different programs were used to apply different classification models: BLOG[40], which is a program specifically developed to handle DNA barcode data, and WEKA [41], which is a software package of machine learning tools for classification, association and clustering problems.

BLOG provides a diagnostic and character-based method, which formulates a set of classification rules that identifies the species in terms of location of key diagnostic nucleotides (*e*.*g*., if position 432 = A, and position 615 = T, then the specimen is classified as *Dalbergia ovata*). BLOG uses the supplied training data set to compute the classification rules, and these rules are then applied on both the training set and the test set, and identification success for both data sets are reported. The seven barcode datasets in this study were tested with a single file input, which was then subject to a 90% slicing, meaning that 90% of the dataset was allocated to training set, and 10% was allocated to test set. The slicing is done within species-level, so for each species, BLOG will allocate 90% of the specimens to the training set, and 10% to the test set.

For identification of the unknown *Dalbergia* spp., all known specimens were included in the training data, and the *Dalbergia* sp. specimens were used as test data.

WEKA in the same way as BLOG works with train and test data either supplied separately or created in the program. WEKA was used to test the four classifiers used by Weitschek *et al*. [43]: Naïve Bayes [66], SMO [67], C4.5 (J48) [68] and Jrip [69]. Naïve Bayes is a Bayesian-based classifier using estimator classes. *A posteriori* probabilities of the species identity are evaluated based on the observed data and *a priori* probabilities [43]. Naïve Bayes does not provide the investigator with a readable model, so the specific assignment of specimens to species cannot be checked manually. SMO is the WEKA version of the function-based method Support Vector Machines (SVM) [67]. SMO converts the reference data into objects in multi-dimensional vectors and then defines an optimal hyperplane that separates the classes with the largest minimum distance. Objects from the query set can then be classified according to the separating hyperplane. SMO usually performs with high classification accuracy, but does not produce a classification model that can be directly read by the investigator [43]. J48 is the WEKA implementation of the decision tree algorithm C4.5 [68], which produces a simple tree structure, where non-terminal nodes represent tests on one or more attributes (here nucleotide type at specific locations). The terminal branches give the results of the decision based on the test. The advantage of the decision-tree method is that the output model is easily read as a set of rules composed of sequence positions and nucleotide compositions. The drawback is that it is very sensitive to variations in the training data[43]. Jrip is the implementation of the rule-based classification method RIPPER (Repeated Incremental Pruning to Produce Error Reduction)[69]. An initial set of rules for each class is generated, and then optimized *k* times. In the same way as BLOG, this method has the advantage that it produces a set of logic rules for each species in the dataset, that can be examined and manually applied to test species if desired [43].

All four classification methods in WEKA were run on all seven data sets, using a single input file and testing with 10-fold cross-validation. For identification of the *Dalbergia* sp., only the SMO classifier was used, as this performed best overall (Table 3). The dataset with *Dalbergia* sp. was loaded as test data set, and a saved model for the SMO and the *rbcL*+*matK*+ITS barcode (*rbcL*+*matK* for the two samples that lacked the ITS sequence) was loaded. The function `re-evaluate model on current test set`was then chosen, selecting outcome predictions as output.

## Results

### Sequences

The *rbcL* region was successfully sequenced for 96 samples, *matK* for 94 samples, while ITS was only sequenced for 79 samples (due to low sequencing success of the herbarium specimens, most likely due to DNA degradation and fungal contamination). All specimens were successfully sequenced for at least two of the three barcoding regions (See S1 Table for details).

For *rbcL*, this resulted in a length of 607 bp, and no length variation was observed for any of the samples.

The *matK* sequences were variable in length, the longest being 851bp, partly due to reading difficulties in both ends of the sequence (primarily because of a long poly-A-sequence). The final alignment was therefore trimmed to 775 bp in order to reduce missing data at the ends. Alignment included one 6-bp insertion for all three *D*. *lanceolaria*-specimen, one 3-bp insertion shared for a group consisting of *D*. *cochinchinensis*, *D*. *ovata* and *D*. *latifolia* specimens, and a 9-bp deletion for the outgroup species *Machaerium lunatum*. All sequences were reverse-complemented to follow standard annotation of the *matK* region.

ITS sequences varied in length, and many sequences were short due to problems with obtaining high quality reads. The ITS5/ITS4 primers used in this study [36] lie in the conserved flanking regions of 18S and 26S, respectively, and the sequences were trimmed to contain only ITS1, 5.8S and ITS2. After trimming, the longest observed sequence was 616 bp for *D*. *cochinchinensis* (628 bp for outgroup *Machaerium*). The alignment including all 79 sequences obtained for ITS was 677 bp and included numerous insertions/deletions. If the removal of specimens from the dataset left blank positions in the alignments because their sequences had insertions not found in any other specimens, these blank positions were removed before further analyses. Excluding the *Machaerium* samples, the alignment used for NJ tree therefore consisted of 666 bp. For the barcoding analysis, where only *Dalbergia* species with at least two specimens where included, the alignment was 662 bp. For the parsimony analysis, including the *Macherium* samples, but excluding the 14 unknown *Dalbergia*, the alignment was 675 bp.

### Taxonomy and phylogenetics of *Dalbergia* species based on parsimony analysis

The *rbcL*+*matK*+ITS dataset for parsimony analysis included 2057 characters, of which 303 were parsimony informative. ITS contributed the most to this number, with 207 informative characters, while *rbcL*+*matK* combined had 96 informative characters.

**Fig 1:**
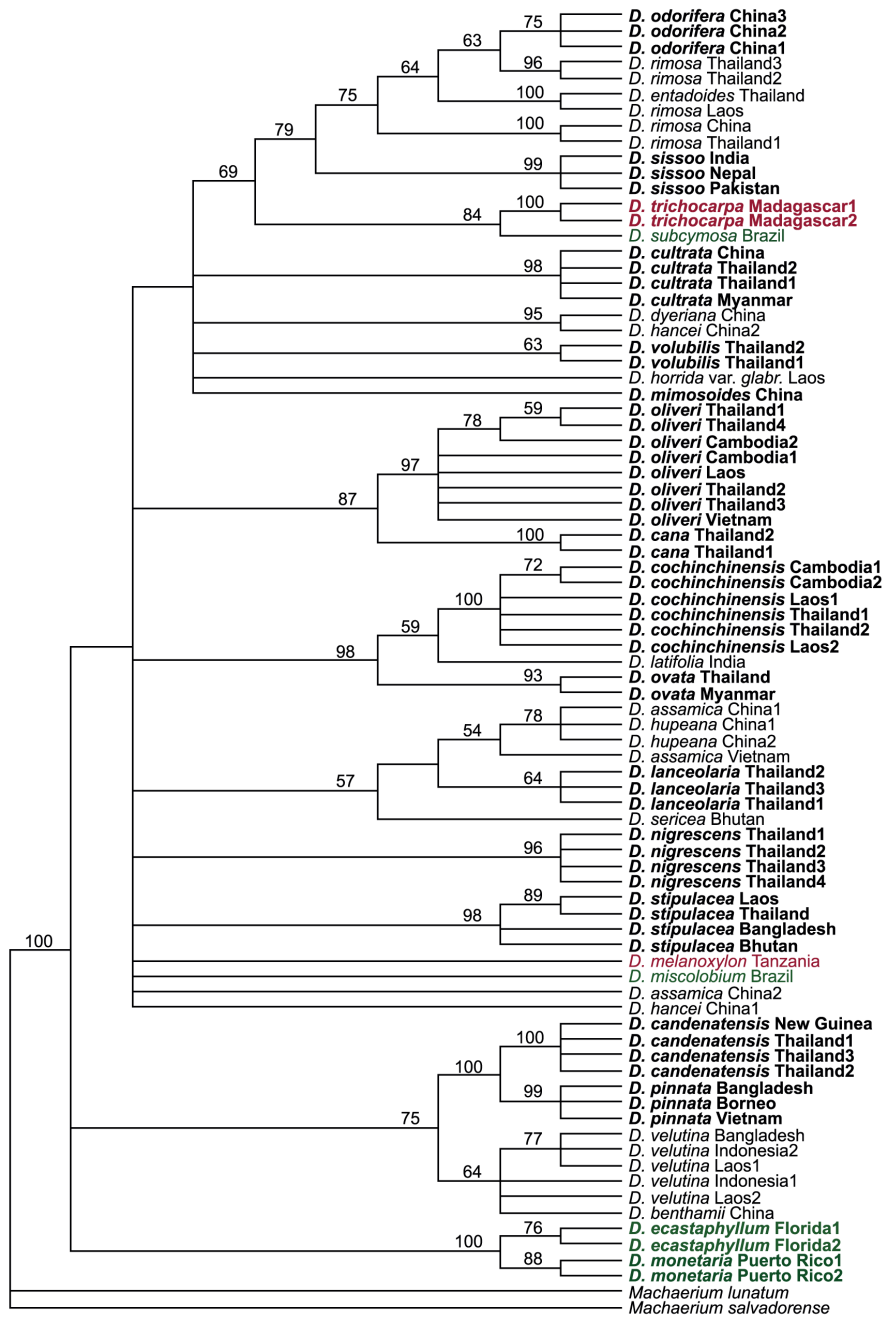
Strict consensus tree resulting from maximum parsimony analysis showing the relationship of *Dalbergia* species using the *rbcL*+*matK*+ITS barcode. Tree length = 906, CI = 0.470, RI = 0.812. Numbers above branches are bootstrap support values; values below 50% are not shown. Monophyletic species with bootstrap values above 60% are given in bold. Species are colored regarding to continent: black: Asia, green: America, red: Africa.

The maximum parsimony analysis resulted in 8,330 equally parsimonious trees, with a tree length of 906 steps. A second round with TBR swapping on these trees produced 15,000 trees, still with a tree length of 906, of which a strict consensus tree was constructed (Fig 1). The consistency index (CI) was 0.470 and the retention index (RI) was 0.812. The strict consensus tree was not well resolved, showing several basal polytomies, which makes inferences on intrageneric relationships difficult. However, a well-supported group consisting of *D*. *candenatensis*, *D*. *pinnata* and *D*. *velutina* (including *D*. *benthamii)* was found at the base of the tree, along with the American species *D*. *ecastaphyllum* and *D*. *monetaria* which were 100% supported as sister species. At the large polytomy, two well-supported groups formed, *D*. *cochinchinensis*, *D*. *ovata* and *D*. *latifolia* (98% bootstrap support), and *D*. *oliveri* together with *D*. *cana* (87%). *Dalbergia subcymosa* was not found to be closely related with the other Brazilian species *D*. *miscolobium*, but to the Malagasy *D*. *trichocarpa*. These two seemed related to a group consisting of *D*. *sissoo* and a *D*. *rimosa/D*. *entadoides/D*. *odorifera-*complex. The resolution was much higher at the tips of the tree. Sixteen of the 21 species represented by two or more specimens were supported as monophyletic with bootstrap values above 60. The two focus species *D*. *cochinchinensis* and *D*. *oliveri*, was found well-resolved with bootstrap values of 100% and 97%, respectively. While *D*. *odorifera* in itself was monophyletic, it was nested within a polyphyletic *D*. *rimosa* group, also containing *D*. *entadoides*. An otherwise well supported cluster of *D*. *velutina* specimens included *D*. *benthamii*, and was therefore not found monophyletic. The two specimens of *D*. *hancei*, which lack ITS sequences, did not cluster together, but were unresolved at the basal polytomy. *D*. *assamica* and *D*. *hupeana* were in the same unresolved group, and could not be separated by the data.

### Distance analysis and barcoding gap for *rbcL*, *matK* and ITS barcodes

Mean intra- and interspecific genetic distances of evaluated DNA barcodes are shown in Table 2.

The *rbcL* region had the lowest values for mean intra- and interspecific distances (*p*-distances of 0.001 and 0.007, respectively), while it was twice as high for the *matK* region, and more than 10-fold higher for ITS. Combined barcodes had intermediate values, where the *rbcL*+*matK* had the lowest values, and the highest values were found for the *rbcL*+*ITS* barcode (probably due to the fact that this was the shortest of the combined regions, and ITS contributed by far the most to the total number of differences between sequences). Four species had no intraspecific variation for the combined *rbcL*+*matK*+ITS barcode (*D*. *cultrata*, *D*. *ecastaphyllum*, *D*. *monetaria* and *D*. *trichocarpa)*. There was an overlap between the observed intra- and interspecific distances for all evaluated barcodes.

**Fig 2:**
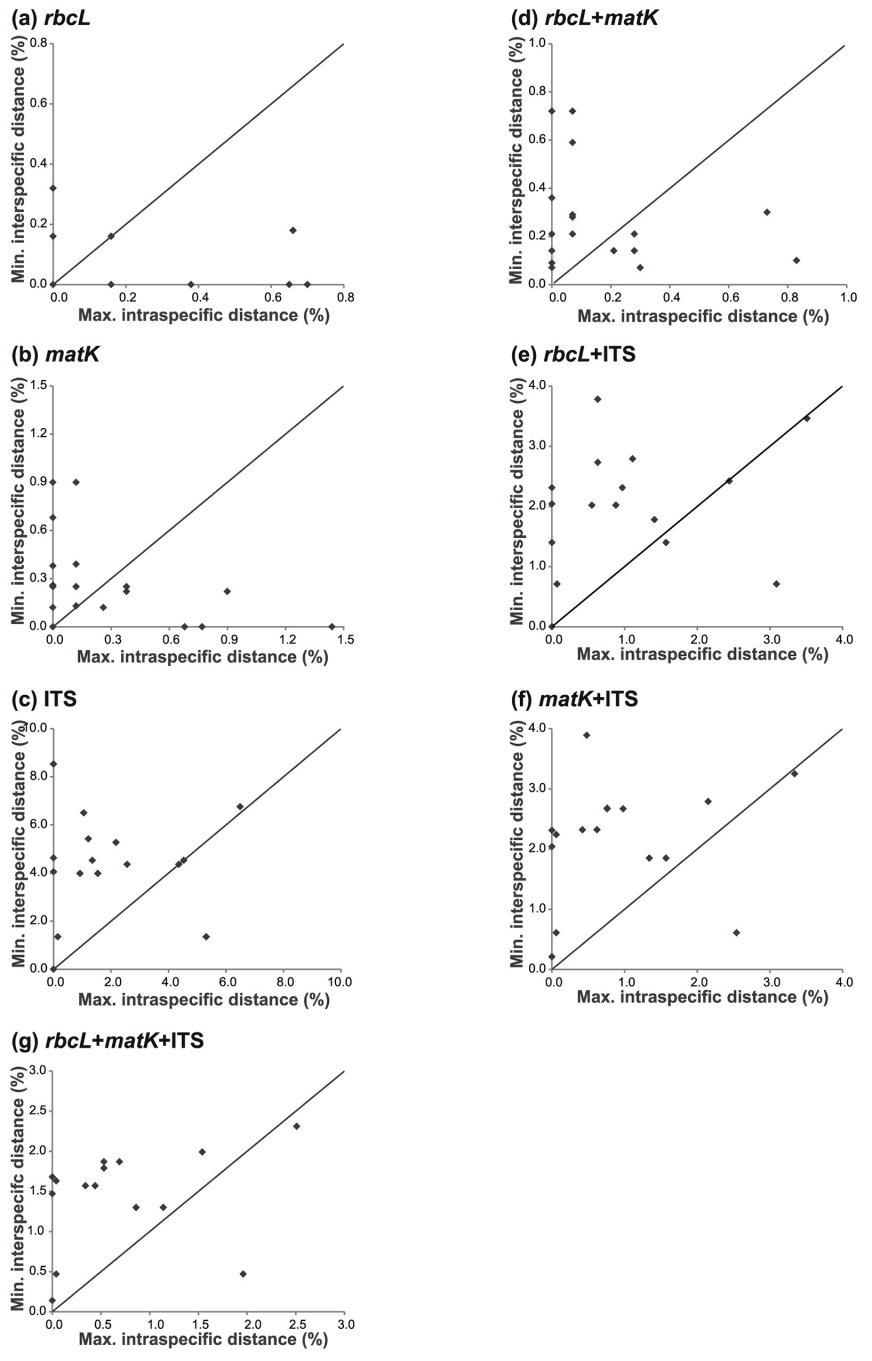
Presence/Absence of barcode gaps in *Dalbergia* spp. for the seven barcodes. Minimum interspecific vs. maximum intraspecific uncorrected *p*-distances (%) for the single (a, b, c) and combined (d, e, f, g) barcodes. Each data point represents one or several species, since some species have identical values of intraspecific and interspecific distances. Species that fall above the 1:1 line exhibit a barcode gap.

Using *rbcL* and *matK* alone, as well as in combination, most species failed to exhibit the barcode gap (Fig 2). The use of ITS greatly improved this picture. The best results were found for the *matK*+ITS barcode, while the added information of *rbcL* did not seem to increase the resolution. *Dalbergia rimosa* and *D*. *velutina* are the two species found under the line for the *matK+*ITS and *rbcL*+*matK*+ITS barcode, which means that no barcoding gap was observed for these species for any barcode.

### Comparison of discrimination methods and barcode regions for specimen identification

We evaluated the discrimination ability of all seven possible combinations of the three barcoding regions included in this study using TaxonDNA, BLOG and four different classification methods implemented in WEKA. The rates of correctly identified, misidentified and not identified specimens for each dataset and method are shown in Table 3.

Averaged over all discrimination methods, the ITS barcode had the highest correct identification rates (78%), and *rbcL* had the lowest (53%). The single highest correct identification rate was obtained for the *matK*+ITS and *rbcL*+*matK*+ITS barcode using TaxonDNA, reaching 100% identification success.

TaxonDNA and SMO were the methods that performed best on average cross all seven tested barcodes (both 86% correct identification), and second-best was the Naïve Bayes method (82%). The rule-based Jrip and the decision tree method J48 generally had low correct identification rates, and especially for the combined barcodes, where the other methods showed high identification rates. TaxonDNA performed better than SMO and Naïve Bayes on the barcodes including the ITS region, however the SMO and Naïve Bayes gave markedly better results on the *rbcL* data set. While barcodes including ITS generally had the highest correct identification rates within each method, BLOG found the highest correct identification rate for *rbcL*+*matK* (86%), and only 65% correct identification for all barcodes involving ITS.

While TaxonDNA and BLOG returned some non-identified results, the four WEKA methods classified all specimens (correctly or wrongly) which meant that the rate of misidentification was relatively lower for TaxonDNA and BLOG than that of the WEKA methods. Thus TaxonDNA showed the average lowest misidentification rates of the six methods (6%), while BLOG had the second lowest at 10% and the SMO method had an average 15% misidentification rate.

### Identification of unknown specimens

The 14 *Dalbergia* sp. specimens were tested against all known *Dalbergia* specimens using TaxonDNA, BLOG and the SMO classifier in WEKA, as well as by examining their position on a NJ tree. While identification in TaxonDNA, SMO and BLOG required several specimens per species, and the reference data therefore only included the 21 species represented by two or more specimens, the NJ tree included all *Dalbergia* species, and thus might give different results.

**Fig 3:**
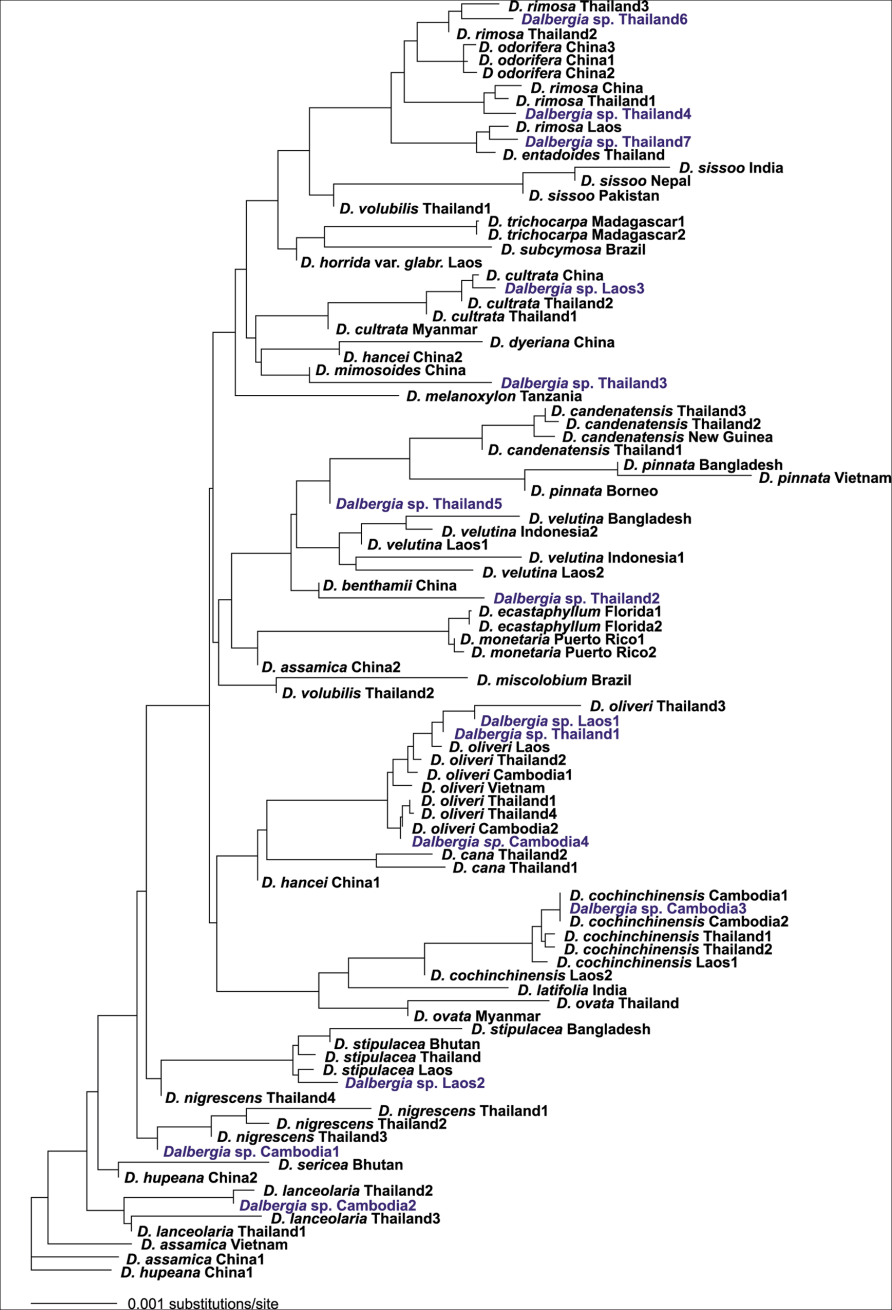
Neighbour-joining tree based on the *rbcL*+*matK*+ITS barcode, including test *Dalbergia* sp. specimens. Uncorrected *p*-distance was used as distance measure.

For nine of the 14 specimens, all four identification methods yielded the same results (Table 4). SMO assigned names to all samples, while BLOG and TaxonDNA did not find any matches for the *Dalbergia* sp. Thailand2 and *Dalbergia* sp. Thailand 3, respectively. The NJ method failed to assign names to five specimens. TaxonDNA, BLOG and SMO returned the same results for the *Dalbergia* sp. Thailand5 and *Dalbergia* sp. Thailand7 (*D*. *velutina* and *D*. *rimosa*, respectively), while no certain identification could be done from the NJ tree (Fig 3). This was most likely due to the presence of *D*. *benthamii* and *D*. *entadoides* in the NJ dataset, which were not included in the barcoding dataset. At the NJ-tree the *Dalbergia* sp. Thailand3 was placed together with *D*. *mimosoides*, but with only one specimen represented, identification cannot be justified.

The cambium/bark and wood samples included in the test of unknown specimens were assigned to *D*. *cochinchinensis* and *D*. *cultrata*, respectively (*Dalbergia* sp. Cambodia3 and *Dalbergia* sp. Laos3, Table 4).

**Table 4:** Identification of *Dalbergia* sp. specimens, based on *rbcL*+*matK*+ITS barcode.

	Tissue	Putative identification	TaxonDNA (within threshold)	NJ-within cluster	BLOG	SMO
***Dalbergia* sp. Cambodia1** ^a^	*Leaf*	*D*. *nigrescens*	*D*. *nigrescens*	*Not identified*	*D*. *nigrescens*	*D*. *nigrescens*
***Dalbergia* sp. Cambodia2**	*Leaf*	*unknown*	*D*. *lanceolaria*	*D*. *lanceolaria*	*D*. *lanceolaria*	*D*. *lanceolaria*
***Dalbergia* sp. Cambodia3**	*Cambium/Bark*	*D*. *cochinchinensis*	*D*. *cochinchinensis*	*D*. *cochinchinensis*	*D*. *cochinchinensis*	*D*. *cochinchinensis*
***Dalbergia* sp. Cambodia4**	*Leaf*	*D*. *oliveri*	*D*. *oliveri*	*D*. *oliveri*	*D*. *oliveri*	*D*. *oliveri*
***Dalbergia* sp. Laos1**	*Leaf*	*D*. *oliveri*	*D*. *oliveri*	*D*. *oliveri*	*D*. *oliveri*	*D*. *oliveri*
***Dalbergia* sp. Laos2**	*Leaf*	*D*. *stipulacea*	*D*. *stipulacea*	*D*. *stipulacea*	*D*. *stipulacea*	*D*. *stipulacea*
***Dalbergia* sp. Laos3**	*Sapwood*	*D*. *cultrata*	*D*. *cultrata*	*D*. *cultrata*	*D*. *cultrata*	*D*. *cultrata*
***Dalbergia* sp. Thailand1**	*Leaf (Herbarium)*	*unknown*	*D*. *oliveri*	*D*. *oliveri*	*D*. *oliveri*	*D*. *oliveri*
***Dalbergia* sp. Thailand2**	*Leaf (Herbarium)*	*unknown*	*D*. *candenatensis*	*Not identified*	*Not classified*	*D*. *pinnata*
***Dalbergia* sp. Thailand3**	*Leaf (Herbarium)*	*unknown*	*outside threshold*	*Not identified*	*D*. *stipulacea*	*D*. *cultrata*
***Dalbergia* sp. Thailand4**	*Leaf (Herbarium)*	*unknown*	*D*. *rimosa*	*D*. *rimosa*	*D*. *rimosa*	*D*. *rimosa*
***Dalbergia* sp. Thailand5** ^a^	*Leaf (Herbarium)*	*unknown*	*D*. *velutina*	*Not identified*	*D*. *velutina*	*D*. *velutina*
***Dalbergia* sp. Thailand6**	*Leaf (Herbarium)*	*unknown*	*D*. *rimosa*	*D*. *rimosa*	*D*. *rimosa*	*D*. *rimosa*
***Dalbergia* sp. Thailand7**	*Leaf (Herbarium)*	*unknown*	*D*. *rimosa*	*Not identified*	*D*. *rimosa*	*D*. *rimosa*

^a^ only *rbcL*+*matK* was used because of lack of amplification success for ITS. NJ = Neighbour Joining. SMO = Support vector machine method, as implemented in WEKA. Putative identification based on morphological characters, except for *Dalbergia* sp. Laos3, where is it based on declared identity from commercial company (See S1 Table for details on origin of samples).

## Discussion

### Taxonomy and phylogeny of South-East Asian *Dalbergia* species

To our knowledge, this analysis represents the first attempt of a molecular phylogeny focused on South-East Asian *Dalbergia*. Vatanparast *et al*. [35] did a molecular phylogeny based on ITS sequences from 64 *Dalbergia* species covering the entire pantropical distribution, but apart from this, classifications within *Dalbergia* are based on morphology [47, 70, 71]. While our data generally gives a high resolution at species level, the basal part of the phylogeny-tree is largely unresolved (Fig 1), making it difficult to evaluate the congruence of molecular data with the morphological classifications. We failed to obtain ITS sequences for specimens of *D*. *benthamii*, *D*. *hancei*, *D*. *horrida*, *D*. *mimosoides* and *D*. *volubilis* and this could be the reason why these specimens are placed at basal polytomies or group with specimens from other species, as ITS in general show the highest discrimination ability (Table 3).

Our analysis only includes few non-Asian species, but the fact that neither the American species nor the African (including Malagasy) species are found together (Fig 1), supports a multiple long-distance dispersal theory as suggested by Vatanparast *et al*. [35].

Although the resolution in the basal parts of the tree is low, a few well-supported groups are seen, which are consistent with findings in previous studies. The apparent sister relationships between *D*. *monetaria* and *D*. *ecastaphyllum* [35, 48] has also been found by Vatanparast *et al*.[35] and Ribeiro [48]. We also find *D*. *pinnata* and *D*. *candenatensis* as sister species, which is consistent with the results by Vatanparast *et al*. [35]. The two latter species are placed as sister to *D*. *velutina* (including one *D*. *benthamii* specimen). This *pinnata/candenatensis/velutina* group is recognized to be morphologically similar by Niyomdham *et al*. [25].

*D*. *cochinchinensis* is highly supported as monophyletic and together with *D*. *latifolia* and *D*. *ovata* forms a well-supported group. Prain [71] placed these three species in the same section, Miscolobium, and Vatanparast *et al*. [35] also found *D*. *cochinchinensis* and *D*. *latifolia* to be closely related. Young individuals of *D*. *cochinchinensis* can be difficult to distinguish from *D*. *ovata* (F. Adema, pers. comm., see also S1 Table), as they have similar leaf morphology, and Niyomdham *et al*. [25] also recognizes the morphological similarity between *D*. *cochinchinensis* and *D*. *ovata*.

We find the timber species *D*. *oliveri* to be well supported as monophyletic. The specimen sampling (n = 8) in this study includes a specimen originally identified as *D*. *dongnaiensis* (*D*. *oliveri* Thailand3, see also S1 Table) and two specimens from Cambodia, where the name *D*. *bariensis* is used. We therefore strongly encourage that the name *D*. *oliveri* is used consistently across the distribution range, as suggested by Niyomdham *et al*. [25].

*Dalbergia oliveri* can be confused with *D*. *lanceolaria* (personal observations), especially if fruits and/or flowers are not present. This study show that barcoding markers can accurately distinguish these two species and that each is supported as monophyletic species (Fig 1). In the analysis of Vatanparast *et al*. [35], a small clade consisting of two *D*. *oliveri*, one *D*. *dongnaiensis* and one *D*. *lakhonensis* is present. According to the Niyomdham *et al*. revision [25], *D*. *dongnaiensis* should be treated as *D*. *oliveri*, and *D*. *lakhonensis* as *D*. *lanceolaria*. Three other *D*. *lanceolaria* specimens in their analysis occur together in another clade, affiliated with *D*. *assamica*/*D*. *balansae*, a relationship we also find (Fig 1). Given the difficulties in species identification in *Dalbergia*, and the number of specimens found to be misidentified in this study alone (S1 Table), it seems possible that the *D*. *lakhonensis* might be a misidentification. If the true identity of this specimen is *D*. *oliveri*, then the Vatanparast *et al*. analysis [35] actually also shows support for the *D*. *oliveri* sensu Nioyomdham.

We treat *D*. *balansae* as a synonym for *D*. *assamica* [25, 49], and the position of *D*. *assamica* China2 is probably due to missing data for ITS. Vatanparast *et al*. [35] maintain *D*. *balansae* as a separate species, but it is found in the same clade as *D*. *assamica*, which could further support the synonymy of *D*. *balansae*.

Missing data could however not account for the fact that *D*. *assamica* and *D*. *hupeana* could not be separated from each other, and that *D*. *rimosa* appeared paraphyletic with both *D*. *entadoides* and *D*. *odorifera* nested within it. It might be due to inadequate resolution of the barcoding data for these taxa, but as the discrimination ability in this study was very high, we find it likely that these results might be indicative of taxonomic uncertainty around these taxa.

We find three separate clades with *D*. *rimosa* specimens, each with bootstrap values of 96 or 100, and one of them including *D*. *entadoides*. This could suggest that *D*. *rimosa* should perhaps be split into several separate species. Further studies including additional DNA regions and a more complete taxon sampling are needed to properly solve these issues, as well as to generally increase the resolution of the phylogeny.

However, we find that the molecular data for the Indochinese species of *Dalbergia* corresponds well with the morphological revision by Niyomdham *et al*. [25], and thus encourage to use this revision for correct naming of species in the Indochina area.

### Which barcoding regions provide the best identification of species in *Dalbergia*?

The barcoding markers generally discriminated well among *Dalbergia* specimens in this study, although the results varied greatly over barcoding regions and analysis methods. We found that ITS had the highest efficiency in identification of specimens in *Dalbergia*, alone or in combination with *matK*. The *rbcL* barcode had the lowest discriminatory power and as a single-locus barcode cannot be recommended for identification of *Dalbergia* specimens. ITS also showed greater ability than *rbcL* and *matK* to produce the `barcode gap’ (Fig 2). The superior discriminatory power of ITS over plastid barcoding regions is consistent with the results of other recent genus-level studies, e.g. for *Euphorbia* [17], *Lamium* [64], *Populus* [72] and *Lysimachia* [73]. However, Yu *et al*. [31] used the chloroplast markers *rpoC*1 and *trnH-psbA* as well as ITS to discriminate between *Dalbergia odorifera* and *D*. *tonkinensis*, and found that *trnH-psbA* discriminated 100% between the two species, whereas ITS and *rpoC*1 yielded inconsistent results.

It should be noted that the analyses for ITS in this study were conducted on a smaller dataset. Four species had only one or no sequences obtained for ITS and thus did not fulfill the criteria for inclusion in barcoding comparisons. Excluding these specimens from the *rbcL*+*matK* analysis as well increases the identification rate from 87% to 92%. It can thus not be ruled out that a smaller data set contributes partly to the difference in identification rates between the *rbcL*+*matK* and ITS regions, an issue experienced in other studies as well [64, 73]. A general concern with the use of ITS is the frequent problems with obtaining high-quality sequences due to low sequence recovery and fungal contaminations [37], which also proved difficult in this study.

Another issue with ITS, and to some extent *matK*, is the challenge with proper sequence alignment. All analysis methods used in this study require aligned sequences, which mean that a new alignment must be made each time a new sequence is introduced to the dataset. Further, alignment of ITS sequences is not straight-forward and most often needs to be manually adjusted after initial alignment with alignment algorithms such as Clustal [54]. A solution could be the application of alignment-free analysis methods, such as BLAST [74] or BRONX [75], but it could also be seen as a reason to choose regions that require no or little alignment work, such as *rbcL* or *matK* in this study.

If applying DNA barcoding for *Dalbergia* as a method of identification of herbarium specimens, or for identification of timber samples for CITES control, the *matK* region, although not as powerful as the ITS for identification, seems a good choice, showing both high sequence recovery and high identification rates. The m*atK* region has proven a useful barcode in other studies of Fabaceae [15, 76], although some report difficulties with amplification and sequencing [77]. The *matK* could be supplemented with *rbcL*, as this seems to increase the discrimination ability slightly (Table 3).

For fresh tissue samples, a two-locus barcode of *matK*+ITS yields optimal discrimination and *rbcL* then seems unnecessary, as it does not enhance the discrimination ability of the data.

As an alternative to the three loci tested in this study, it might be worth considering the intergenic spacer *trnH-psbA*. Generally high discrimination ability of this marker have been acknowledged by the CBOL Plant Working group [12] and Yu *et al*. [31] found it to be efficient on two *Dalbergia* species, why it could be worth testing on a larger sample of *Dalbergia* species.

### Which analytic methods provide the best identification of species in *Dalbergia*?

TaxonDNA and SMO were the two methods that gave the highest correct identification rates; however TaxonDNA had the single highest rates and SMO performed better at the *rbcL* dataset with low variation. The decision-tree method J48 and the rule-based Jrip did not yield impressing results for any of datasets. Weitchek *et al*. [43] also found J48 and Jrip to be less efficient than the other machine learning methods, although not as low performing as in this study. Comparisons between the different identification methods used in this study should however be interpreted with caution, as the sampled number of specimens per species is not optimal and the different methods have not been tested in the same way. While the four WEKA classification methods were tested with 10-fold cross-validation, the Taxon-DNA is a nearest-neighbour method, and is tested with leave-one-out validation (LOO). Cross-validation or LOO is currently not implemented in BLOG, and the 90% / 10% testing of the data is sensitive to data containing species represented by only two specimens. In fact, species represented only by two specimens were involved in the majority of the misidentifications and none-identifications in BLOG, for the analysis including ITS (*D*. *assamica*, *D*. *cana*, *D*. *ecastaphyllum*, *D*. *lanceolaria*, *D*. *monetaria*, *D*. *nigrescens*). For BLOG and the WEKA classification methods, the authors recommend at least four specimens per species for optimal performance. As this criterion is only met for a few species in this study, our dataset might not give justice to these methods, and it might be part of the explanation why the Jrip and J48 methods show low performance across datasets. The rule-based BLOG also does not seem very well suited for imperfect datasets as the present, as “a complete reference library of polymorphisms for each species is required in the training set to avoid false negatives” [40]. This is a general weakness of the method, as it is not always possible to sample all variation within the species, and also not necessary if other methods are capable of handling such variation, such as TaxonDNA or the SMO function. The problem with species only represented by two specimens, as well as the fact that any ITS sequence in the test set is likely to harbor variation not seen in the train set, probably explain why BLOG has low identification success for any barcode combination including ITS (65%, Table 3).

For TaxonDNA, it cannot be ruled out that the low number of specimens in some species could possibly cause an underestimation of intraspecific distances. If including more specimens to the dataset would result in higher maximum intraspecific distances, this would reduce the barcoding gap, and perhaps also affect discrimination rates by the Best Close Match method. Whether any effect would be positive or negative would dependent on chance and how well the total intraspecific variation is already covered in the dataset.

The potential bias by sample sizes set aside, our results seem to support TaxonDNA as a highly workable and accurate method. TaxonDNA is very simple to use and understand, and has been accepted as a standard method to evaluate barcodes in many studies (e.g. [17, 64, 73, 78–80]).

An optimal DNA barcode classifier would not only yield high correct identification rates, but should also show a minimum level of misidentifications. As reference databases are rarely perfect in real situations, ability to detect specimens not covered in the reference database and return “not identified” results is a desirable feature for a DNA barcode classifier. This aspect favours TaxonDNA, as well as BLOG, over the WEKA classification methods as they are currently implemented. However, if dealing with a dataset with low variation, our data indicates that the Naïve Bayes or SMO method might be used with greater success rates than TaxonDNA. Under some circumstances, BLOG could be the method of choice, as it produces a set of rules to characterize each species in terms of nucleotides at particular positions. This feature might be desirable *e*.*g*. in designing species-specific assays to be used in CITES enforcement.

### Efficiency of the DNA barcodes to identify unknown *Dalbergia* specimens

We found that the different methods generally converge on their results on identification on unknown specimens, and the putative ID (based on morphological observations) for six of the samples were confirmed. So, can we trust the proposed identity of the specimens using these barcoding markers? Including the high discrimination results found in this study, the answer would be “yes”. However, rather than expecting the barcoding data to unravel the universal truth, it should be regarded critically, the quality of the reference database needs to be taken into account, and the data seen as a supplement to morphological identification.

The NJ tree has a few more non-identified or ambiguous results, which is probably due to the fact that more species were included in the NJ tree than in the other analyses. This stresses the importance of adequate taxon sampling: not including all relevant species in the reference database can lead to possible misidentifications. This is particularly true for the SMO method, which assigned names to all the query sequences. TaxonDNA shows the advantage of a “not identified” category, as it does not identify specimens if the distance to the nearest reference-sequence is outside the threshold computed for that particular dataset. This could prevent many cases of misidentifications, and instead highlight issues with missing taxa in the reference database. For the specimens that were not classified or had divergent results, it could help guide the morphological identification process, and also serve as valuable reference data which might lead to identification once the database is augmented.

### Application of barcoding tools in conservation of *Dalbergia*

Barcoding tools can support on-going conservation measures of the species in several ways. Species delimitation and identification is the first critical step in an accurate assessment of distribution, population abundance and threats of target species. In the present study we *e*.*g*. found that the *rbcL*+*matK*+ITS barcode markers accurately identified *D*. *oliveri* as monophyletic in accordance with Niyomdham *et al*. (1997) [25]. Hence, the IUCN red list assessment for this species can now be updated, including the data from the synonyms *D*. *bariensis*, *D*. *mammosa*, and *D*. *dongnaiensis*, to gain a more accurate assessment of the conservation status of this species.

The application of DNA methods to verify species identity and origin of internationally traded timber has attracted increasing interest in recent years as part of global systems to support sustainable forestry and especially reduce illegal logging [81, 82]. Several studies has shown ability of DNA barcoding to effectively discriminate threatened from common species, such as among *Euphorbia* species from Madagascar [17], and among timber species in the mahogany family (Meliaceae) [18]. Yu *et al*. [31] showed that barcoding markers had the potential to discriminate between the precious timber species *D*. *odorifera* and the closely related, but less valuable species *D*. *tonkinensis*.

Enhancing the applicability for direct use in CITES enforcement, genus or species-specific assays independent on expensive sequencing and reference databases can be developed. Examples of specific assays that have been developed include a probe-based real-time PCR assay targeted to discriminate between *Gonostylus* and non-CITES genera in Thymeleaceae [83], and a PCR/restriction enzyme assay to discriminate between *Swietenia* and non-CITES genera in Meliaceae [84]. Specific assays can also target shorter fragments of DNA, which increases the probability of amplification of degraded DNA from wood samples [85].

Illegal logging represents a huge threat to the *Dalbergia* timber species in Indochina. Given the high discrimination success rates found in this study, *Dalbergia* seems an ideal candidate for using DNA barcoding for identification of traded timber. The present study provides a database to which sequences from new samples could be matched, as well as necessary sequences from which a species-specific assay for testing of *D*. *cochinchinensis*/not *D*. *cochinchinensis* could be designed. Such an assay could be used in CITES enforcement and also in local control measures. This study was able to identify wood samples which shows that DNA barcoding has the potential to be applied as a timber identification tool in *Dalbergia*, although it is likely that the process stage of the timber will have an influence on the ability to extract DNA samples of adequate quality, as well as whether the samples are sapwood or heartwood [31, 85, 86].

Future floristic investigations can benefit from the reference database established for *Dalbergia* in Indochina. We suggest future botanical collections in the region to include leaf samples in silica gel, for subsequent DNA extraction, sequencing and comparison to the reference database. Presently, DNA facilities are not available in national herbaria in the region, but due to the simplicity of the barcoding approach the DNA work can easily be outsourced at moderate costs. The obtained sequences can be matched to the database by herbarium staff. At first, the database will have significant amounts of missing data, but this problem will decrease as the use of this method increases and the population of verified sequences grow. Local herbaria have only recently been revitalized after years of political unrest in the region and often suffer from shortage of staff. Barcoding can speed up the identification process of collections, including sterile material, and increase knowledge on species distributions and abundance. This will allow more detailed assessment of threatened species that can guide conservation efforts and priorities at national and regional levels.

## Supporting Information

S1 Table Voucher information and Genbank accession numbers for the 93 *Dalbergia* and two *Machaerium* specimens included in the present study. (DOCX)

S2 Table Corrections of identification for *Dalbergia* herbarium specimens. (DOCX)

S3 Table Primers used for amplification and sequencing in DNA barcoding of *Dalbergia*. (DOCX)

S4 Table Detailed results of identification success of *Dalbergia* specimens using the `best match’ and `best close match’ methods in TaxonDNA/SpeciesIdentifier 1.7.7. (DOCX)
