# Supplementary material for: The Use of DNA Barcoding in Identification and Conservation of Rosewood (Dalbergia spp.)
Source: PLoS One. 2015 Sep 16;10(9):e0138231. doi: 10.1371/journal.pone.0138231 (PMC4573973; doi:10.1371/journal.pone.0138231)
Supplement: S1 Table — (DOCX) [file pone.0138231.s001.docx]

S1 Table. Voucher information and Genbank accession numbers for the 93 *Dalbergia* and two *Machaerium* specimens included in the present study.

| **Specimen name, incl. origin** | **Voucher** | **Genbank accession numbers** | | |
| --- | --- | --- | --- | --- |
|  |  | **rbcL** | **matK** | **ITS** |
| *D. assamica* China1 | Hu & But 22805 (L) | KM510234 | KM521275 | KM521354 |
| *D. assamica* China2 | Fei-yan *et al.* 1530a (AAU) | KM510233 | KM521273 | - |
| *D. assamica* Vietnam | Poilane 25005 (AAU) | KM510231 | KM521272 | KM521356 |
| *D. benthamii* China | Hu & But 20533 (L) | KM510238 | KM521278 | - |
| *D. cana* Thailand1 | FRDU & Van der Welzen 9 (L) | KM510244 | KM521284 | KM521358 |
| *D. cana* Thailand2 | Larsen 2260 (AAU) | KM510239 | KM521279 | KM521357 |
| *D. candenatensis* New Guinea | Verdcourt & Katik 4964 (L) | KM510243 | KM521280 | KM521359 |
| *D. candenatensis* Thailand1 | Kerr 15404 (AAU) | KM510240 | KM521281 | KM521360 |
| *D. candenatensis* Thailand2 | Van der Kevie 25 (L) | KM510242 | KM521283 | KM521362 |
| *D. candenatensis* Thailand3 | Larsen 30989 (AAU) | KM510241 | KM521282 | KM521361 |
| *D. cochinchinensis* Cambodia1 | Hartvig Larsen 28 (C) | KM510248 | KM521288 | KM521363 |
| *D. cochinchinensis* Cambodia2 | Virachey Botanical Survey 114 (E) | KM510246 | KM521286 | KM521364 |
| *D. cochinchinensis* Laos1 | Hartvig Larsen 35 (C) | KM510249 | KM521289 | KM521365 |
| *D. cochinchinensis* Laos2 | Nanthavong BT499 (E) | KM510287 | KM521326 | - |
| *D. cochinchinensis* Thailand1 | Premradsamee 46 (C) | KM510245 | KM521285 | KM521366 |
| *D. cochinchinensis* Thailand2 | Middleton D.J. 233 (K) | KM510247 | KM521287 | KM521367 |
| *D. cultrata* China | M. Czako, Univ. South Carolina, USA (coll. from Xishuangbanna Tropical Botanical Garden, Yunnan, China) | KM510260 | KM521300 | KM521369 |
| *D. cultrata* Myanmar | Flora of Burma 4141, Herb. J.H. Lace (K) | KM510252 | KM521292 | - |
| *D. cultrata* Thailand1 | Niyomdham *et al.* 130 (C) | KM510250 | KM521290 | KM521371 |
| *D. cultrata* Thailand2 | Hartvig Larsen & Maxwell 37 (C) | KM510251 | KM521291 | KM521370 |
| *D. dyeriana* China | Sino-America Guizhou Botanical Expedition 2221 (L) | KM510255 | KM521295 | KM521372 |
| *D. ecastaphyllum* Florida1 | living coll., M. Czako, Univ. South Carolina, USA | KM510256 | KM521296 | KM521373 |

Continued

S2 Table. Continued

| **Specimen name, incl. origin** | **Voucher** | **Genbank accession numbers** | | |
| --- | --- | --- | --- | --- |
|  |  | **rbcL** | **matK** | **ITS** |
| *D. ecastaphyllum* Florida2 | living coll., M. Czako, Univ. South Carolina, USA | KM510257 | KM521297 | KM521374 |
| *D. entadoides* Thailand | Niyomdham 5099 (AAU) | KM510258 | KM521298 | KM521375 |
| *D. hancei* China1 | Leeuwenberg 14069 (L) | KM510262 | KM521303 | - |
| *D. hancei* China2 | Hu & But 21333 (L) | KM510263 | KM521302 | - |
| *D. horrida* var. *glab.* Laos | Poilane 11947 (AAU) | KM510264 | KM521304 | - |
| *D. hupeana* China1 | Wang Yang-Ming 326 (K) | KM510265 | KM521305 | KM521378 |
| *D. hupeana* China2 | Lei 913 (L) | KM510266 | KM521306 | - |
| *D. lanceolaria* Thailand1 | Maxwell 00-194 (CMU) | KM510268 | KM521307 | - |
| *D. lanceolaria* Thailand2 | living coll., M. Czako, Univ. South Carolina, USA | KM510229 | KM521270 | KM521379 |
| *D. lanceolaria* Thailand3 | Niyomdham 894 (C) | KM510269 | KM521308 | KM521380 |
| *D. latifolia* India | living coll., M. Czako, Univ. South Carolina, USA | KM510270 | KM521309 | KM521381 |
| *D. melanoxylon* Tanzania | living coll., M. Czako, Univ. South Carolina, USA | KM510271 | KM521310 | KM521382 |
| *D. mimosoides* China | F 138 (AAU) | KM510272 | KM521311 | - |
| *D. miscolobium* Brazil | living coll., M. Czako, Univ. South Carolina, USA | KM510273 | KM521312 | KM521383 |
| *D. monetaria* Puerto Rico1 | living coll., M. Czako, Univ. South Carolina, USA | KM510274 | KM521313 | KM521384 |
| *D. monetaria* Puerto Rico2 | living coll., M. Czako, Univ. South Carolina, USA | KM510275 | KM521314 | KM521385 |
| *D. nigrescens* Thailand1 | Larsen 3344 (L) | KM510278 | KM521317 | KM521386 |
| *D. nigrescens* Thailand2 | living coll., M. Czako, Univ. South Carolina, USA | KM510279 | KM521318 | KM521387 |
| *D. nigrescens* Thailand3 | Larsen 31702 (AAU) | KM510276 | KM521315 | - |
| *D. nigrescens* Thailand4 | Larsen 34002 (AAU) | KM510277 | KM521316 | - |
| *D. odorifera* China1 | M. Czako, Univ. South Carolina, USA (coll. from Xishuangbanna Tropical Botanical Garden, Yunnan, China) | KM510281 | KM521320 | KM521389 |
| *D. odorifera* China2 | Chun, N.K. & C.L. Tso 44712 (K) | KM510280 | KM521319 | KM521388 |

Continued

S2 Table. Continued

| **Specimen name, incl. origin** | **Voucher** | **Genbank accession numbers** | | |
| --- | --- | --- | --- | --- |
|  |  | **rbcL** | **matK** | **ITS** |
| *D. odorifera* China3 | How 70769 (K) | KM510261 | KM521301 | KM521377 |
| *D. oliveri* Cambodia1 | Hartvig Larsen 21 (C) | KM510237 | KM521277 | KM521390 |
| *D. oliveri* Cambodia2 | Hartvig Larsen 30 (C) | KM510236 | KM521276 | KM521391 |
| *D. oliveri* Laos | Hartvig Larsen 36 (C) | KM510286 | KM521325 | KM521392 |
| *D. oliveri* Thailand1 | Niyomdham 915 (C) | KM510282 | KM521321 | KM521393 |
| *D. oliveri* Thailand2 | Thai-Leiden Expedition 1973 5841 (E) | KM510284 | KM521323 | KM521394 |
| *D. oliveri* Thailand3 | Bloembergen 528 (K) | - | KM521294 | KM521395 |
| *D. oliveri* Thailand4 | Larsen 2153 (L) | KM510254 | KM521324 | KM521396 |
| *D. oliveri* Vietnam | Hartvig Larsen 13 (C) | KM510283 | KM521322 | KM521397 |
| *D. ovata* Myanmar | Po Khant 1084 (K) | KM510288 | KM521327 | - |
| *D. ovata* Thailand | Maxwell 75-159 (L) | KM510289 | KM521328 | KM521398 |
| *D. pinnata* Bangladesh | Huq & A.I. 10430 (L) | KM510291 | KM521330 | KM521399 |
| *D. pinnata* Borneo | Lugas 1843 (K) | KM510290 | KM521329 | KM521400 |
| *D. pinnata* Vietnam | Cuong et al 1045 (L) | KM510292 | - | KM521401 |
| *D. rimosa* China | M. Czako, Univ. South Carolina, USA (coll. from Xishuangbanna Tropical Botanical Garden, Yunnan, China) | KM510230 | KM521271 | KM521402 |
| *D. rimosa* Laos | Libman & Souliya LAOS_628 (L) | KM510294 | KM521333 | KM521403 |
| *D. rimosa* Thailand1 | Larsen 45463 (L) | KM510293 | KM521332 | KM521404 |
| *D. rimosa* Thailand2 | living coll., M. Czako, Univ. South Carolina, USA | KM510295 | KM521334 | KM521405 |
| *D. sericea* Bhutan | Grierson & Long 1711 (K) | KM510297 | KM521336 | KM521406 |
| *D. rimosa* Thailand3 | Bjørnland/Schumacher 570 (C) | KM510259 | KM521299 | KM521376 |
| *D. sissoo* India | Hiremath 111 (K) | KM510300 | - | KM521407 |
| *D. sissoo* Nepal | Herbal Garden, Kanazawa Univ. Japan (East Nepal) 9555013 (E) | KM510299 | KM521338 | KM521408 |

Continued

S2 Table. Continued

| **Specimen name, incl. origin** | **Voucher** | **Genbank accession numbers** | | |
| --- | --- | --- | --- | --- |
|  |  | **rbcL** | **matK** | **ITS** |
| *D. sissoo* Pakistan | Rechinger 29498 (AAU) | KM510298 | KM521337 | KM521409 |
| *D. stipulacea* Bangladesh | Huq & A.I. 10437 (L) | KM510267 | - | KM521410 |
| *D. stipulacea* Bhutan | Grierson & Long 3555 (K) | KM510303 | KM521341 | KM521411 |
| *D. stipulacea* Laos | Newman *et al.* LAO 322 (E) | KM510302 | KM521340 | KM521412 |
| *D. stipulacea* Thailand | Larsen 2995 (C) | KM510301 | KM521339 | KM521413 |
| *D. subcymosa* Brazil | living coll., M. Czako, Univ. South Carolina, USA | KM510304 | KM521342 | KM521414 |
| *D. trichocarpa* Madagascar1 | living coll., M. Czako, Univ. South Carolina, USA | KM510305 | KM521343 | KM521415 |
| *D. trichocarpa* Madagascar2 | living coll., M. Czako, Univ. South Carolina, USA | KM510306 | KM521344 | KM521416 |
| *D. velutina* Bangladesh | Huq & Mia 10336 (L) | KM510296 | KM521335 | KM521417 |
| *D. velutina* Indonesia1 | Argent & Wilkie 941 (E) | KM510307 | KM521345 | KM521418 |
| *D. velutina* Indonesia2 | Kessler et al BK2022 (L) | KM510310 | KM521348 | KM521419 |
| *D. velutina* Laos1 | Newman et al LAO 302 (E) | KM510308 | KM521346 | - |
| *D. velutina* Laos2 | Svengsuksa BT 334 (L) | KM510309 | KM521347 | KM521420 |
| *D. volubilis* Thailand1 | Niyomdham *et al.* 1326 (AAU) | KM510312 | KM521350 | - |
| *D. volubilis* Thailand2 | Maxwell 74-142 (AAU) | KM510311 | KM521349 | - |
| *Machaerium lunatum* | living coll., M. Czako, Univ. South Carolina, USA | KM510313 | KM521425 | KM521421 |
| *Machaerium salvadorense* | living coll., M. Czako, Univ. South Carolina, USA | KM510315 | KM521352 | KM521422 |
| *Dalbergia sp.* Cambodia1 | Hartvig Larsen 42 (C) | KM676468 | KM676478 | - |
| *Dalbergia sp*. Cambodia2 | Hartvig Larsen 41 (C) | KM676476 | KM676487 | KM676496 |
| *Dalbergia sp.* Cambodia3 | Hartvig Larsen 44 (Univ. Copenhagen, Denmark) | KP719018 | KP719020 | KP719022 |
| *Dalbergia sp.* Cambodia4 | Hartvig Larsen 29 (C) | KP719017 | KP719019 | KP719021 |
| *Dalbergia sp*. Laos1 | Hartvig Larsen 38 (C) | KM676477 | KM676488 | KM676497 |

Continued

S2 Table. Continued

| **Specimen name, incl. origin** | **Voucher** | **Genbank accession numbers** | | |
| --- | --- | --- | --- | --- |
|  |  | **rbcL** | **matK** | **ITS** |
| *Dalbergia sp.* Laos2 | Hartvig Larsen 39 (C) | KM676467 | KM676479 | KM676489 |
| *Dalbergia sp.* Laos3 | Amazon Exotic Hartwoods | KM510253 | KM521293 | KM521368 |
| *Dalbergia sp*. Thailand1 | Nielsen 1565 (AAU) | KM676469 | KM676480 | KM676490 |
| *Dalbergia sp.* Thailand2 | Larsen 42720 (AAU) | KM676471 | KM676482 | KM676492 |
| *Dalbergia sp.* Thailand3 | Larsen 43011 (AAU) | KM676472 | KM676483 | KM676493 |
| *Dalbergia sp*. Thailand4 | Larsen 43544 (AAU) | KM676473 | KM676484 | KM676494 |
| *Dalbergia sp.* Thailand5 | Larsen 43549 (AAU) | KM676475 | KM676485 | - |
| *Dalbergia sp.* Thailand6 | Larsen 44537 (AAU) | KM676474 | KM676486 | KM676495 |
| *Dalbergia sp.* Thailand7 | Pooma 5178 (AAU) | KM676470 | KM676481 | KM676491 |

Letters in brackets correspond to herbarium codes, L = Nationaal Herbarium Nederland, Leiden University branch, AAU = Aarhus University, Denmark,
C = Natural History Museum of Denmark, Copenhagen, E = Royal Botanic Garden Edinburgh, UK, K = Royal Botanic Gardens, Kew, UK.

”-” means sequencing failed.
