# Supplementary material for: The Use of DNA Barcoding in Identification and Conservation of Rosewood (Dalbergia spp.)
Source: PLoS One. 2015 Sep 16;10(9):e0138231. doi: 10.1371/journal.pone.0138231 (PMC4573973; doi:10.1371/journal.pone.0138231)
Supplement: S2 Table — (DOCX) [file pone.0138231.s002.docx]

**S2 Table. Corrections of identification for *Dalbergia* herbarium specimens.**

| **Initial identification** | **Voucher information** | **Corrected identification**  **(as used in analyses)** | **Note** |
| --- | --- | --- | --- |
| ***D. ovata* Graham ex Benth.** | Nanthavong BT499 (L) | ***D. cochinchinensis* Pierre** | Misidentification, correction accepted by L (F. Adema, pers. comm.,) |
| ***D. sericea* G. Don /*burmanica* ? /*velutina* Benth.** | Huq & Mia 10336 (L) | ***D. velutina* Benth.** | Uncertain identification corrected, correction accepted by L (F. Adema, pers. comm.) |
| ***D. lanceolaria*** | Huq & A.I. 10437 (L) | ***D. stipulacea* Roxb.** | Misidentification, correction accepted by L (F. Adema, pers. comm.) |
| ***D. dongnaensis* Pierre** | Blombergen 528 (K) | ***D. oliveri* Gamble ex Prain** | Synonym [[1](#_ENREF_1)] |
| ***D. hainanensis*** **Merr. & Chun** | How 70769 (K) | ***D. odorifera* Chen** | Misidentification, duplicates identified as *D. odorifera* [[2](#_ENREF_2), [3](#_ENREF_3)] |
| ***D. balansae* Prain** | Fei-yan et al 1530a (AAU) | ***D. assamica* Benth.** | Synonym [[1](#_ENREF_1), [4](#_ENREF_4)] |
| ***D. balansae* Prain** | Hu & But 22805 (L) | ***D. assamica* Benth.** | Synonym [[1](#_ENREF_1), [4](#_ENREF_4)] |
| ***D. foliacea* Wall. Ex. Benth** | Bjørnland/Schumacher 570 (C) | ***D. rimosa* Roxb.** | Synonym [[5-7](#_ENREF_5)] |

1. Niyomdham C, Hô PH, Dy Phon P, Vidal JE, editors. Leguminoseae-Papilionoideae Dalbergieae. Paris: Muséum National d'Histoire Naturelle; 1997.

2. *Dalbergia odoriferia* How 70769 at the Herbarium at the Institute of Botany Database Beijing, China: Chinese Academy of Sciences; [24 November 2014]. Available from: <http://www.nhpe.org/pe/00177180>.

3. *Dalbergia odorifera* How 70769 at the Chinese Virtual Herbarium (CVH) [24 November 2014]. Available from: <http://www.cvh.org.cn/db/data_allspmfinal/data_pview.php?id=IBK_IBK00071099>.

4. Chen D, Zhang D, Larsen K. Tribe Dalbergieae. In: Wu ZY, Raven PH, Hong DY, editors. Flora of China, Volume 10 (Fabaceae). Beijing, China and St. Louis, USA: Science Press and Missouri Botanical Garden Press; 2010.

5. The Plant List Version 1.1 (2013) [15 June 2015]. Available from: <http://www.theplantlist.org/>.

6. Thothathri K. Taxonomic revision of the tribe Dalbergieae in the Indian subcontinent. Calcutta, India: Botanical survey of India; 1987.

7. Gagnepain F. Dalbergia. In: Lecomte MH, editor. Flore Générale de l´IndoChine. 2. Paris, France: Masson et Cie 1913.
