# Supplementary material for: The Use of DNA Barcoding in Identification and Conservation of Rosewood (Dalbergia spp.)
Source: PLoS One. 2015 Sep 16;10(9):e0138231. doi: 10.1371/journal.pone.0138231 (PMC4573973; doi:10.1371/journal.pone.0138231)
Supplement: S3 Table — (DOCX) [file pone.0138231.s003.docx]

S3 Table. Primers used for amplification and sequencing in DNA barcoding of *Dalbergia.*

| **Region** | **Primer name** | **5´- 3´ sequence** | **References** |
| --- | --- | --- | --- |
| **rbcL** | rbcl_AF | ATGTCACCACAAACAGAGACTAAAGC | Kress & Erickson [[1](#_ENREF_1)] |
|  | Ajf634R | GAAACGGTCTCTCCAACGCAT | Fazekas *et al.* [[2](#_ENREF_2)] |
| **matK** | matK-3F_IH | CGTATAGTACTCTTGTGTTTACGAG | This study, modified from Ki-Joong Kim, unpublished [[3](#_ENREF_3)] |
|  | matK-1R_IH | ATCCTATTCATCTGGAAATCTTGGTTC | This study, modified from Ki-Joong Kim, unpublished [[3](#_ENREF_3)] |
| **ITS** | ITS5 | GGAAGGAGAAGTCGTAACAAGG | China Plant BOL Group [[4](#_ENREF_4)] |
|  | ITS4 | TCCTCCGCTTATTGATATGC | China Plant BOL Group [[4](#_ENREF_4)] |

1. Kress WJ, Erickson DL. A Two-Locus Global DNA Barcode for Land Plants: The Coding rbcL Gene Complements the Non-Coding trnH-psbA Spacer Region. Plos One. 2007;2(6):e508.

2. Fazekas AJ, Burgess KS, Kesanakurti PR, Graham SW, Newmaster SG, Husband BC, et al. Multiple Multilocus DNA Barcodes from the Plastid Genome Discriminate Plant Species Equally Well. Plos One. 2008;3(7):e2802.

3. Kim K-J. School of Life Sciences and Biotechnology, Korea University, Seoul, Korea.

4. Li DZ, Gao LM, Li HT, Wang H, Ge XJ, Liu JQ, et al. Comparative analysis of a large dataset indicates that internal transcribed spacer (ITS) should be incorporated into the core barcode for seed plants. P Natl Acad Sci USA. 2011;108(49):19641-6.
