# Supplementary material for: The Use of DNA Barcoding in Identification and Conservation of Rosewood (Dalbergia spp.)
Source: PLoS One. 2015 Sep 16;10(9):e0138231. doi: 10.1371/journal.pone.0138231 (PMC4573973; doi:10.1371/journal.pone.0138231)
Supplement: S4 Table — (DOCX) [file pone.0138231.s004.docx]

S4 Table. Detailed results of identification success of *Dalbergia* specimens using the ´best match´ and ´best close match´ methods in TaxonDNA/SpeciesIdentifier 1.7.7 [[1](#_ENREF_1)].

| **Barcode** | **Threshold** | **Best match** | | | **Best close match** | | | |
| --- | --- | --- | --- | --- | --- | --- | --- | --- |
|  |  | Succesfully identified (%) | Ambiguous | Misidentified | Succesfully identified (%) | Ambiguous | Misidentified | No match |
| ***rbcL*** | 0.0052 | 28 (40.0%) | 38 (54.28%) | 4 (5.71%) | 28 (40.0%) | 38 (54.28%) | 4 (5.71%) | 0 (0.0%) |
| ***matK*** | 0.0064 | 55 (80.88%) | 4 (5.88%) | 9 (13.23%) | 55 (80.88%) | 4 (5.88%) | 9 (13.23%) | 0 (0.0%) |
| **ITS** | 0.0583 | 50 (89.28%) | 6 (10.71%) | 0 (0.0%) | 50 (89.28%) | 6 (10.71%) | 0 (0.0%) | 0 (0.0%) |
| ***rbcL*+*matK*** | 0.0057 | 58 (86.56%) | 2 (2.98%) | 7 (10.44%) | 58 (86.56%) | 2 (2.98%) | 7 (10.44%) | 0 (0.0%) |
| ***rbcL*+ITS** | 0.0317 | 49 (89.09%) | 4 (7.27%) | 2 (3.63%) | 49 (89.09%) | 4 (7.27%) | 2 (3.63%) | 0 (0.0%) |
| ***matK*+ITS** | 0.0290 | 53 (100.0%) | 0 (0.0%) | 0 (0.0%) | 53 (100.0%) | 0 (0.0%) | 0 (0.0%) | 0 (0.0%) |
| ***rbcL*+*matK*+ITS** | 0.0216 | 52 (100.0%) | 0 (0.0%) | 0 (0.0%) | 52 (100.0%) | 0 (0.0%) | 0 (0.0%) | 0 (0.0%) |

1. Meier R, Shiyang K, Vaidya G, Ng PKL. DNA Barcoding and Taxonomy in Diptera: A tale of High Intraspecific Variability and Low Identification Success. Syst Biol. 2006;55(5):715-28.
